# A systematic review and future directions for AI-driven detection of fraud patterns in SACCO transactions

**DOI:** 10.3389/frai.2025.1690482

**Published:** 2026-01-30

**Authors:** Dalton Ampumuza, Calorine Katushabe, Micheal Tamale

**Affiliations:** Department of Computer Science, Faculty of Computing and Information Science, Kabale University, Kabale, Uganda

**Keywords:** artificial intelligence, financial transactions, fraud detection, fraud patterns, machine learning, PRISMA, SACCO

## Abstract

Fraud in Savings and Credit Cooperative Organizations (SACCOs) remains a major challenge that undermines financial inclusion and sustainability in developing countries. This study conducted a systematic literature review to examine both traditional and emerging fraud patterns and evaluate fraud detection methods with emphasis on artificial intelligence and machine learning applications. A comprehensive structured search across Web of Science, Scopus, and Google Scholar yielded 28 peer-reviewed studies published between 2015 and 2025 that met eligibility and quality criteria. The findings reveal that traditional fraud patterns such as member collusion, embezzlement, and asset misappropriation coexist with emerging digital fraud such as mobile payment fraud, phishing, card fraud, and cryptocurrency scams. While rule-based and audit-based detection remain ineffective, machine learning has demonstrated significant promise for real-time detection but faces challenges related to class imbalance, interpretability, and data privacy. The review identified a weak Information and Communication Technology (ICT) infrastructure, the absence of SACCO-specific fraud detection models, and hybrid frameworks. It concludes that hybrid models that integrate traditional audit methods with machine learning are recommended for SACCO-specific fraud detection frameworks. This study emphasizes the need for future research on explainable AI and privacy-preserving analytics to enhance fraud resilience in SACCOs.

## 1 Introduction

Savings and Credit Cooperative Societies (SACCOs) play a central role in financial inclusion and socioeconomic development in many developing countries. These constitutions provide accessible credit, promote savings, support micro-entrepreneurs, and remain crucial for employment and poverty reduction (Kembo and Mwakujonga, 2013). For example, in 2023, Kenyan SACCOs contributed 45% to the country’s Growth Domestic Product (GDP), while in Tanzania, they contributed approximately 40%. These SACCOs also absorb a significant proportion of new graduates into the workforce (Kembo and Mwakujonga, 2013), demonstrating their importance to the growth of the private sector (Zhu, 2021).

Despite their benefits, SACCOs face persistent fraud risks that undermine performance, weaken institutional credibility, lead to financial losses, and, in severe cases, may even cause collapse. Fraud often involves embezzlement, collusion, loan diversion, and manipulation of financial statements. The Association of Certified Fraud Examiners (ACFE) defines fraud as any scheme or activity that relies on one’s deception to gain something. Existing literature on fraud remains descriptive, focusing on isolated cases rather than synthesizing systematic fraud patterns, detection methods, or emerging digital threats (Mumanyi, 2014).

### 1.1 Global evolution of fraud and its relevance to SACCOs

Globally, financial institutions (FIs) are experiencing unprecedented fraud challenges that are driven by rapid digital transformations. As digital banking expands, fraud schemes involving flash fraud, phishing, malware, cryptocurrency scams, and account-takeover attacks have also become more sophisticated. Traditional fraud detection strategies, largely manual and rule-based, are no longer adequate for modern threats that require real-time monitoring, behavioral analytics, and adaptive learning systems (Kembo and Mwakujonga, 2013; Ojino and Ndolo, 2023a). While these trends are well-documented in banks and large financial institutions, their implications for SACCOs are less understood. SACCOs are increasingly digitizing their operations; however, they often lack advanced detection capabilities. This creates vulnerabilities where both traditional and technologically enabled fraud schemes can coexist.

### 1.2 Purpose of the study

Existing artificial intelligence and machine learning research on fraud focuses predominantly on credit card fraud, online banking, and digital payments (Yuhertiana and Amin, 2024; Akinsola, 2025). Limited attention has been given to the SACCO-specific environment, despite its unique operational structure, heterogeneous data formats, and operational dynamics. There is also a lack of a hybrid model that combines traditional audit mechanisms with advanced machine learning tailored to SACCO operations.

This review addresses these gaps by:

1. Identifying traditional and emerging fraud patterns that affect SACCOS.
2. Examining existing fraud detection methods.
3. Proposing a hybrid AI-based framework suitable for low-resource financial settings such as SACCOs.

## 2 Methodology

This review was conducted following the Preferred Reporting Items for Systematic Reviews and Meta-Analyses (PRISMA) guidelines. This study sought to answer the following three questions:

1. What traditional and emerging fraud patterns affect SACCOs?
2. Which fraud detection methods have been applied, and how effective are they in the SACCO context?
3. What gaps exist in current detection approaches, and what does AI/ML offer for SACCO-specific fraud detection?

### 2.1 Scope of the study

The researcher focused on peer-reviewed studies (journal articles, conference papers, and systematic literature reviews) published between 2015 and 2025. The literature directly addresses SACCOs and fraud detection in comparable financial institutions.

Non-scholarly sources, such as newspapers, blogs, opinion articles, and non-peer-reviewed reports, were excluded.

### 2.2 Search strategy and screening

A comprehensive search was conducted across different databases such as Web of Science, Scopus, and Google Scholar using a search string that included Boolean combinations “AND” “OR,” such as SACCO Fraud “AND” fraud detection, cooperative fraud detection AND machine learning and digital fraud, “OR” financial fraud.

**Figure 1:**
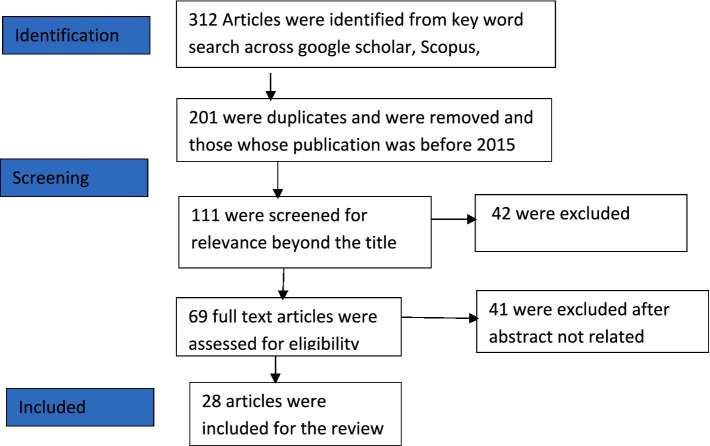
PRISMA flow (source: Authors’ own work).

The initial search yielded 312 articles. After screening for relevance, duplicates, and out-of-scope publications, 69 articles remained. The final study included 28 articles after full-text assessment (see Figure 1).

## 3 Findings

This systematic literature review opens a discussion on the different patterns highlighted by recent studies and examines the proposed solutions.

### 3.1 Taxonomy of fraud patterns

SACCOs can be categorized into traditional and emerging patterns, each driven by distinct socio-technical and organizational dynamics (Harris et al., 2013). Traditional patterns predominantly result from internal collusions and weak governance systems, such as financial statement manipulation, asset misappropriation, corruption, and bribery, which are often facilitated by manual systems, limited information and communication technology (ICT) tools, and a high reliance on trust-based operations.

Emerging fraud patterns are technology-driven fraud schemes that involve a shift from human-based manipulation to cyber-enabled deception and increased complexity, such as mobile payment fraud with malicious applications to intercept or divert funds, card fraud, identity theft, phishing, cryptocurrency, and investment fraud (Yuhertiana and Amin, 2024).

Various approaches have been adopted to overcome these problems. Traditional approaches such as auditing, forensic accounting, and rule-based systems are reactive in nature and often fail to detect evolving fraud (Akinsola, 2025). AI-driven machine learning approaches, such as supervised, unsupervised, and hybrid machine learning, have proven successful and scalable, despite the challenges of class imbalance and privacy concerns.

**Table 1:** 

Paper/Study	Fraud pattern(s)	Fraud detection	Author
Knowledge Graph for fraud detection: Case of fraudulent transactions Detection in Kenyan SACCO	Corruption, asset misappropriation, and fraudulent financial statements	The paper identifies current fraud patterns in SACCO transactions. It proposed an approach that detects and prevents transaction risks by leveraging knowledge graphs	Ojino (2023)
Fraud detection in financial transactions	Imbalanced datasets, privacy and security concerns, and low latency in transaction processing.	The paper does not specifically address current fraud patterns in SACCO transactions. However, it highlights that financial fraud is evolving with technology, and machine learning can analyze transaction patterns to detect anomalies	Dama (2024)
Financial Fraud Prevention Through Strengthened Corporate Governance: A Discourse	Identity theft and phishing	The increasing sophistication of fraud in financial systems due to digitization emphasizes the need for advanced cybersecurity measures and machine learning techniques for stakeholders to protect the interests of their customers	Vishva (2024), Bamigboye (2020)
Fraud Detection and Prevention in Financial Institutions	Credit card fraud	Credit card fraud is the most commonly addressed fraud type using machine learning techniques. The review emphasizes challenges such as class imbalance in datasets and the dynamic nature of fraud patterns, suggesting that these issues may also be relevant to SACCO transactions, indicating a need for further research in this area.	Lamgade (2024)
Model based on clustering and association rules for the detection of fraud in banking transactions	Small transaction fraud	This paper introduces a semi-supervised model combining both clustering and association rules to detect fraud in banking transactions, while emphasizing the importance of analyzing customer behavior patterns to improve detection accuracy while minimizing false positives	Mehrdad Kargari (2018)
A comprehensive survey on fraud detection methods in financial transactions	Transactional fraud	Shreenidhi reviews various fraud detection methods applicable to financial transactions that include machine learning, data mining, and a rule-based approach, emphasizing the integration of AI and real-time analytics to enhance detection accuracy and adaptive learning for the evolving fraud patterns	Ti (2024)
Regulatory Compliance and the Role of Corporate Governance in Preventing Financial Misstatements	Regulatory and compliance failures with financial stress	Regulatory frameworks that are weak allow corporate fraud to infiltrate the systems; propose stronger compliance and transparent operations by authenticating financial reports. The study further highlights that pressure on management to meet extreme targets can trigger fraudulent activities such as fraudulent reporting; however, structures to mitigate such pressure have also been proposed.	Akinsola (2025)
The roles of the whistleblowing system and fraud awareness as a financial statement fraud deterrent	Underreporting and whistleblowing	Nanang reported that employees feared retaliation and were reluctant to report, proposed that whistleblowing systems are key informants for fraud detection alongside a corporate culture that encourages reporting suspicious activities, and stated that there is a need to increase awareness in human capital.	Shonhadji (2021)
Preventing financial statement fraud in the corporate sector: insights from auditors	Manipulation of financial statements to deceive stakeholders regarding a company’s financial position	This paper proposes to strengthen internal controls and regular audits with proactive fraud risk assessments while underscoring the effectiveness of these internal controls	Mandal (2023), Mega et al. (2024)
Intelligent financial fraud detection practices in the post-pandemic era	Digital fraud schemes on online transactions and account impersonations are influenced by the increasing use of digital means	This paper proposed the use of artificial intelligence and data analytics for real-time transaction monitoring to effectively detect fraudulent transactions	Zhu (2021)

Fraud in financial statements is another key area of concern. A study titled *Preventing Financial Statement Fraud in the Corporate Sector: Insights from Auditors* proposes strengthening internal controls and conducting regular audits alongside proactive fraud risk assessments. These measures are suggested to effectively prevent the manipulation of financial information; the study further recognizes the role of whistleblowing systems and fraud awareness in deterring fraud activities by auditors (Mandal, 2023; Mega et al., 2024).

The study by Shonhadji (2021) on under-reporting emphasizes that many employees are reluctant to report suspicious activities due to fear of retaliation; it concludes that whistleblowing systems play a vital role in fraud detection and a supportive corporate culture is necessary to encourage reporting while increasing awareness among staff.

Finally, the study *Intelligent Financial Fraud Detection in the Post-Pandemic Era* addresses emerging digital fraud schemes, such as online transaction manipulation and account impersonation, which have become more common with the adoption of digital technologies. This study recommends leveraging artificial intelligence and the integration of different techniques, such as multiple models or data analytics, for real-time transaction monitoring to effectively detect and prevent fraudulent activities (Zhu, 2021).

### 3.2 Discussion

This systematic review reveals that fraud in SACCOs is multifaceted, encompassing both traditional and emerging forms of digital fraud enabled by technological integration. The reviewed literature indicates that fraud often results from weak internal controls, behavioral motivations, and the rapid digitization of financial services, which has not been matched by adequate cybersecurity measures (Akinsola, 2025; Mega et al., 2024; Lamgade, 2024).

### 3.3 Fraud patterns

The persistence of internal collusion-based fraud remains a dominant issue in the operations of SACCO. Studies such as that by Odeyemi et al. (2024) have highlighted that fraudulent practices often involve embezzlement, financial statement manipulation, and asset misappropriation by staff or board members who exploit governance and member trust.

In addition, digital transformation has introduced new vulnerabilities. As SACCOs adopt mobile payment systems and third-party integration services, they become susceptible to technology-induced fraud, such as phishing, card skimming, identity theft, and account takeover (Ojino and Ndolo, 2023b; Alhchaimi, 2024). This evolving attack reflects a shift from manual manipulation of records to computer-related deception. Unlike traditional fraud, which can be uncovered through audits, digital fraud requires advanced analytics, anomaly detection, and behavioral profiling techniques.

Furthermore, the review underscores the behavioral and ethical dimensions. Yuhertiana and Amin (2024) argued that organizational culture and ethical standards have a direct influence on fraud prevalence. Weak ethical leadership, financial pressure, and rationalization are key components in fraud training and remain critical explanatory variables for the persistent fraud in SACCOs.

### 3.4 Effectiveness of AI fraud detection techniques

A comparative analysis of detection methods indicates that artificial intelligence-based and hybrid detection methods outperform traditional rule-based mechanisms in identifying complex fraud patterns (Yuhertiana and Amin, 2024). Traditional approaches, including auditing and forensic accounting, are important for governance; however, they are largely reactive and dependent on manual reviews. As a result, they are incapable of handling the growing transaction volumes in digitized SACCO environments (Yuhertiana and Amin, 2024; Dama, 2024).

In contrast, machine learning and artificial intelligence have demonstrated superior performance in the prediction and detection of fraudulent activities with the use of models and algorithms such as XGBoost, Random Forest, and neural networks, which can learn behavioral patterns and flag anomalies in real time (Alhchaimi, 2024; Kajal and Kaur, 2021). Hybrid systems that combine rule-based and machine learning methods have shown improved precision and reduced false positives (Yuhertiana and Amin, 2024). However, their deployment in SACCOs remains minimal due to institutional and infrastructure barriers, including limited access to labelled datasets, insufficient ICT infrastructure, and skilled personnel, to develop and maintain these AI systems.

Supervised Learning Models require labeled datasets containing known fraud and legitimate cases (Kajal and Kaur, 2021). However, these datasets may be limited in SACCO environments. Random Forest remains one of the most widely applied models due to its resilience against noise, ability to model non-linear relationships, and effectiveness in handling structured financial data. Studies demonstrate that Random Forest achieves strong performance in detecting credit card, mobile banking, and insider fraud in the financial ecosystem (Kajal and Kaur, 2021; Dama, 2024).

Extreme Gradient Boosting (XGBoost) and Artificial Neural Networks (ANNs) have been dominant due to their ability to handle imbalanced datasets, whereas ANNs address the behavioral context and non-linear patterns (Seify et al., 2022). Although these have demonstrated superior performance, they often fall short of transparency and interpretability challenges, especially in regulated environments, such as SACCOs, where auditability and explainability are essential (Jurgovsky et al., 2018).

### 3.5 Unsupervised and semi-supervised approaches

Given that SACCOs often lack labelled fraud datasets, unsupervised and semi-supervised approaches such as clustering, autoencoders, and rule-based approaches are highly applicable. Clustering algorithms such as K-means detect fraudulent activities by identifying outliers that deviate from typical transactions and detecting unusual relationships that may indicate collusion, among others (Vishva, 2024).

Hybrid and Ensemble Models integrate rule-based with machine learning for improved accuracy and reduced false positives. Hybrid architectures benefit from the interpretability of rule-based detection and adaptive learning capabilities. Studies have shown that combining deterministic and probabilistic methods significantly enhances detection.

### 3.6 Gap

Despite increasing scholarly attention to financial fraud detection, a notable gap remains in SACCO-specific frameworks. The majority of existing AI and machine learning models are adapted from banking or credit card fraud studies and fail to account for the unique governance structures, heterogeneous data formats, and behavioral dynamics of SACCOs [24, 25]. Furthermore, few studies have provided quantitative evidence of the effectiveness of AI-driven approaches in SACCO settings, highlighting the need for context-specific validation.

Concerns regarding data privacy, availability, and quality concerns are prevalent, as SACCOs often lack integrated systems capable of capturing and labeling fraudulent transactions, thereby limiting opportunities for supervised machine learning. Consequently, unsupervised anomaly detection remains promising but underexplored in SACCO fraud analytics (Mehrdad Kargari, 2018).

### 3.7 Framework

To overcome the limitations of fraud detection in low-resource environments such as SACCOs with AI and evolving methods, this study proposes a framework that incorporates both traditional and digital forms of fraud detection. AI-driven fraud detection systems differ from traditional rule-based systems in that they learn from transaction patterns, identify hidden correlations, and adapt to new fraud patterns in real time. The proposed framework builds on the understanding that SACCOs operate in a heterogeneous, data-constrained environment and are characterized by a limited ICT infrastructure. Within the detection layer, several algorithms are used in combination with the traditional rule-based approach to classify fraudulence to ensure both interpretability and precision in fraud detection.

**Figure 2:**
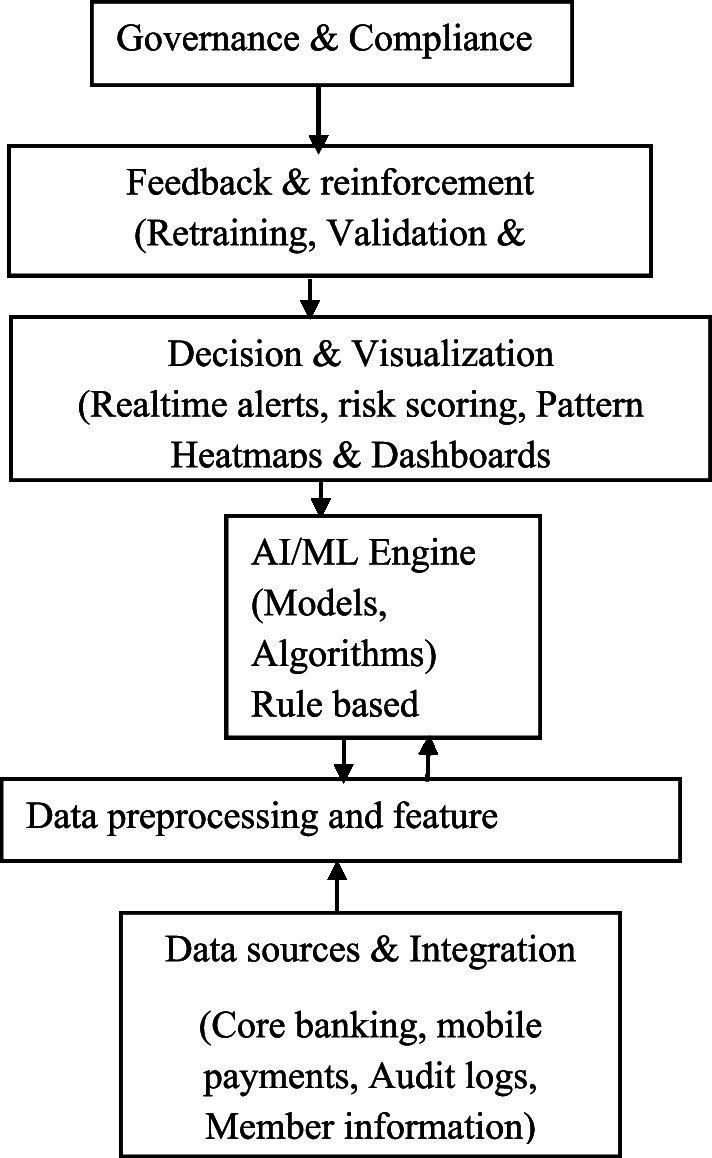
Framework for AI adoption in fraud detection in low-resource settings like SACCOs.

The subsequent layers of the framework support decision-making, learning, and governance to ensure the continuous adaptability and ethical use of artificial intelligence. The decision support translates analytical results into actionable insights through fraud risk scoring, real-time alerts, and visualization dashboards to aid SACCO staff in prioritizing investigations. The governance and compliance layers embed explainable AI to guarantee transparency, data confidentiality, and adherence to regulatory standards (see Figure 2).

## 4 Conclusion and recommendations

The findings of this study reveal that fraud in SACCOs is multifaceted, with both traditional and emerging digital fraud patterns. Traditional fraud patterns remain deeply rooted within SACCO operations and are primarily driven by weak internal controls, overreliance on trust, and technological gaps, whereas emerging digital fraud is becoming more prevalent as SACCOs embrace digital platforms without corresponding cyber capacity. This convergence of traditional and digital fraud underscores the need for integrated and adaptive detection systems (Mehrdad Kargari, 2018; Alhchaimi, 2024).

Furthermore, it was demonstrated that artificial intelligence and machine learning-based detection techniques outperform traditional and rule-based approaches in detecting complex, real-time anomalies. Machine learning models such as Random Forest, XGBoost, and neural networks show significant potential in analyzing large transaction datasets, evolving techniques, and minimizing false positives (Kajal and Kaur, 2021; Akinsola, 2025). However, adoption remains limited because of the challenges faced by SACCOs. The results, therefore, affirm the inadequacy of standalone traditional methods and advocate for hybrid frameworks that integrate AI analytics with manual oversight to achieve effective detection. Consistent with the need to review the critical gap, the absence of SACCO-specific fraud detection models, inadequate data governance frameworks, and limited validation of AI techniques within the SACCO context makes the use of AI and machine learning underexplored despite their potential for detecting fraud.

Therefore, this study concludes that the future of fraud detection in SACCOs lies in the continuous improvement of the framework and development of context-aware hybrid AI models that combine traditional, behavioral analytics, and automated mechanisms while advancing the design of explainable AI systems tailored to SACCO environments to ensure transparency, trust, and interpretability of detection results.
